# Synthesis and determination of the absolute configuration of (−)-(5*R*,6*Z*)-dendrolasin-5-acetate from the nudibranch *Hypselodoris jacksoni*

**DOI:** 10.3762/bjoc.9.329

**Published:** 2013-12-23

**Authors:** I Wayan Mudianta, Victoria L Challinor, Anne E Winters, Karen L Cheney, James J De Voss, Mary J Garson

**Affiliations:** 1 School of Chemistry and Molecular Biosciences, The University of Queensland, Brisbane QLD 4072, Australia; 2 School of Biological Sciences, The University of Queensland, Brisbane QLD 4072, Australia

**Keywords:** enantioselective HPLC, *E*/*Z*-isomers, *Hypselodoris*, natural products, nudibranch, sesquiterpene

## Abstract

A small sample of (−)-(5*R*,6*Z*)-dendrolasin-5-acetate, which was fully characterized by 2D NMR studies, was isolated from the nudibranch *Hypselodoris jacksoni*, along with the sesquiterpenes (+)-agassizin, (−)-furodysinin, (−)-euryfuran, (−)-dehydroherbadysidolide and (+)-pallescensone. A synthetic sample ([α]_D_ −8.7) of the new metabolite was prepared by [1,2]-Wittig rearrangement of a geranylfuryl ether followed by acetylation of purified alcohol isomers. The absolute configuration at C-5 was established as *R* by the analysis of MPA ester derivatives of (*Z*)-5-hydroxydendrolasin obtained by preparative enantioselective HPLC.

## Introduction

Marine organisms have been proven a prolific source of natural products that potentially can be used as lead compounds or which have inspired synthetic chemists as synthetic targets [1]. Similarly, extensive studies of marine chemical ecological interactions have enriched our knowledge of biological and evolutionary patterns, and of the role of small molecules in chemical defensive strategies underwater [2–3]. Nudibranchs (Mollusca: Gastropoda: Opisthobranchia) are a group of marine animals that use small molecules for a variety of purposes, including communication, reproduction, and defense against predation [4–5]. These molecules have been extensively studied and as of 2012, seven out of the 20 natural products of marine origin (either directly or as a derivative) that have been approved for FDA use as a drug or are in clinical trials were first isolated from molluscs. For some of these bioactives, the actual biosynthetic source is a microorganism [6].

**Figure 1:**
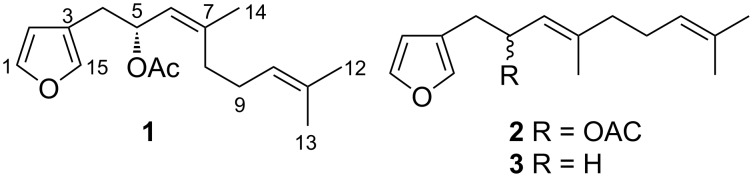
(−)-(5*R*,6*Z*)-Dendrolasin-5-acetate (**1**), its 6*E* derivative and naturally occuring dentrolasin (**3**).

Within the Nudibranchia, the genus *Hypselodoris* is generally characterized by sesquiterpene chemistry, as evidenced by a number of studies on European [7–13], Indian [14], South African [15], Brazillian [16], and Indo-Pacific specimens [17–20]. During a comparative biogeographical study on opisthobranch molluscs from South East Queensland, Australia, we encountered the new sesquiterpene (−)-(5*R*,6*Z*)-dendrolasin-5-acetate (**1**) in specimens of *H. jacksoni* along with the other known sesquiterpene metabolites (+)-agassizin, (−)-furodysinin, (−)-euryfuran, (−)-dehydroherbadysidolide, (+)-pallescensone. The new metabolite **1** has never been identified from natural sources, although the 6*E*-isomer **2** was previously reported in racemic form during the synthesis of naturally occurring dendrolasin (**3**) by Tsubuki et al. in 2009 [21] (Figure 1). Dendrolasin (**3**) itself has been isolated from both terrestrial and marine sources, including from the nudibranchs *Chromodoris lochi* [22], from three *Hypselodoris* spp. [18], and from the marine sponge *Dictyodendrilla* sp. [23]. The current study reports the isolation and synthesis of (−)-(5*R*,6*Z*)-dendrolasin-5-acetate (**1**), with the determination of the absolute configuration at C-5 by spectroscopic analysis of the methoxyphenylacetic acid (MPA) derivative.

## Results and Discussion

### Isolation and structure elucidation of natural product **1**

Eight individuals of *H. jacksoni* were collected by SCUBA from two locations in SE Queensland. Owing to their small size (21–40 mm), the specimens were not examined individually. Extraction into acetone followed by partitioning into diethyl ether gave an extract which was fractionated first by normal phase (NP) flash chromatography and then by preparative NP HPLC to give (+)-agassizin, (−)-furodysinin, (−)-euryfuran, (−)-dehydroherbadysidolide, (+)-pallescensone, and the new sesquiterpene **1**.

**Figure 2:**
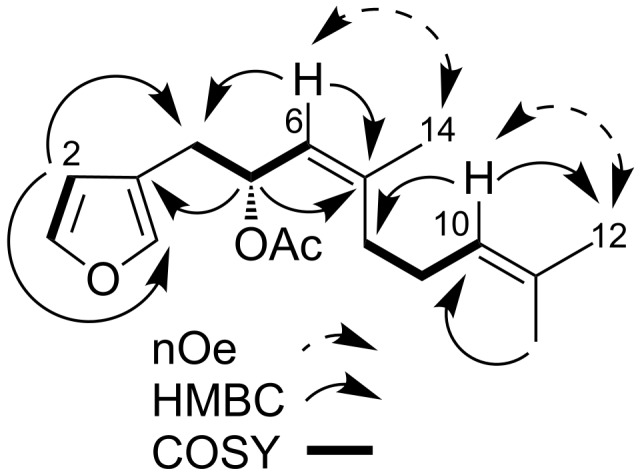
Selected HMBC, COSY, and nOe correlations of **1**.

Compound **1**, [α]_D_ −53 (*c* 0.006, CHCl_3_), displayed a (+)-HRESIMS ion adduct at *m*/*z* 299.1624 [M + Na]^+^ consistent with the molecular formula C_17_H_24_O_3_. The ^1^H NMR spectrum (CDCl_3_, 500 MHz) of **1** showed diagnostic signals for a furan [δ_H_ 7.33 (1H), 7.23 (1H), 6.27 (1H); δ_C_ 142.7, 140.1, 111.8] along with two alkene signals [δ_H_ 5.15 (1H), 5.07 (1H); δ_C_ 123.7, 123.9]. Signals for three methyl groups were observed at δ_H_ 1.72, 1.67, and 1.59 and were linked to signals at δ_C_ 23.3, 25.7, and 17.6, respectively, by HSQC. There were also signals characteristic of an acetate group [δ_H_ 2.01 (s, 3H); δ_C_ 21.4]. These partial NMR assignments were comparable to those of the known dendrolasin (**3**), a sesquiterpene previously isolated from *Hypselodoris cantabrica* [9] and *H. californiensis* [18]. However, the signal corresponding to H-5 in dendrolasin (**3**) was reported to be a multiplet (2H) resonating at δ_H_ 2.21 [24] whereas that in **1** was shifted to δ_H_ 5.64 (brdt, *J* = 6.4, 9.3 Hz, 1H). The gCOSY spectrum of **1** showed cross peaks from H-5 to H-4/H-6, between the furan signals H-1/H-2 and between H-8/H-9/H-10. The H-5 proton was attached to an oxymethine carbon at δ_C_ 70.7 based on HSQC data and showed HMBC correlations to neighbouring carbons including C-3 (δ_C_ 120.2), C-7 (δ_C_ 141.5), and the OAc carbonyl (δ_C_ 170.2) (Figure 2). HMBC correlations observed from the alkene proton H-6 (δ_H_ 5.15) to C-4, C-7, and CH_3_-14 secured the position of the double bond between C-6 and C-7. A second double bond was positioned between C-10 and C-11 based on HMBC correlations from the alkene proton H-10 (δ_H_ 5.07) to CH_3_-12 and C-8. The C-6/C-7 configuration was revealed as *Z* rather than the *E*-configuration anticipated, based on 1D nOe experiments in which the signal intensity of H_3_-14 increased when the signal attributed to H-6 was irradiated (mixing time 60 ms). 1D nOe irradiation of H-10 provided assignment of the signals corresponding to CH_3_-12.

### Total synthesis of (−)-(5*R*,6Z)-dendrolasin-5-acetate (**1**)

**Scheme 1:**
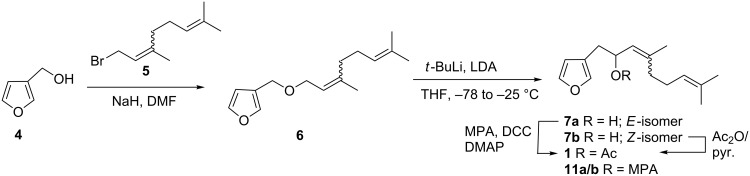
Synthetic route to sesquiterpene **1**.

In view of the small sample (0.1 mg) of natural material available, a synthetic study was undertaken to provide stereochemical characterization of **1**, in particular the configuration at C-5. The synthesis, which was completed in five steps, involved preparation of 3-furylmethanol (**4**) and of geranyl bromide (**5**), etherification, [1,2]-Wittig rearrangement of geranyl 3-furylmethyl ether (**6**) to produce **7a/b**, and acetylation to obtain target **1**. The overall synthesis (Scheme 1) was adapted from Tsubuki et al. [21].

Furan-3-ylmethanol (**4**) was prepared in 78% yield by reduction of 2-furoic acid with LiAlH_4_ in dry diethyl ether, whereas geranyl bromide (**5**) was obtained through bromination of geraniol with carbon tetrabromide and triphenylphosphine in 90% yield. The ^1^H NMR spectrum of the geraniol sample used indicated the presence of traces of nerol, the *Z*-isomer of geraniol. Etherification of furan-3-ylmethanol (**4**) with the geranyl/neryl bromide mixture (**5**) in the presence of NaH furnished the 3-furylmethyl ether **6** (65% yield) as an *E*/*Z* mixture (3:1). The H-5 signal for the minor *Z-*isomer of **6** resonated at δ_H_ 3.90 (d, *J* = 6.8 Hz) compared to that of the *E*-isomer (δ_H_ 4.00, d, *J* = 6.8 Hz). Irradiation of the H-5 signal of the *Z*-isomer increased the intensity of the signal associated with the adjacent methyl (CH_3_-14), confirming the Z double bond at C6/C7. The isomeric mixture of **6** was immediately subjected to [1,2]-Wittig rearrangement with *t*-BuLi (4 equiv) and LDA at −78 °C to afford racemic 5-hydroxydendrolasin (39% yield), again as an *E*/*Z* mixture (**7a/b**) for which a 3:1 ratio was determined by ^1^H NMR comparison with data for the individual stereoisomers (see below). The 3:1 ratio of *E*/*Z-*isomers was confirmed via analytical HPLC.

**Scheme 2:**
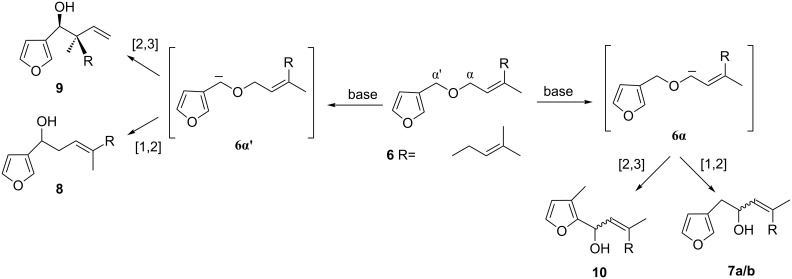
Mechanism of Wittig rearrangement [21].

In principle, the rearrangement of **6** could afford either [1,2]-rearranged products (e.g. **7a/b** or **8**) or [2,3]-rearranged products such as **9** or **10** depending on which site adjacent to the ether oxygen is preferentially deprotonated (Scheme 2). A thermodynamic optimization study on the Wittig rearrangement of allyl 3-furylmethyl ether by Tsubuki et al. has revealed that deprotonation occurs mainly at the α-position [21]. The results of this theoretical study are fully consistent with our synthetic work since the mixture of **7a/b** was the sole [1,2]-Wittig rearrangement product obtained.

**Figure 3:**
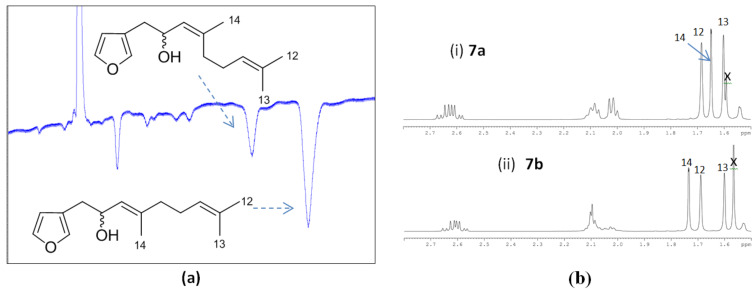
(a) NP HPLC trace showing separation of racemic 5-hydroxydendrolasin (**7a/b**) using hexanes/EtOAc (90:10; flow rate 2 mL/min over 60 minutes (x axis)) and refractive index detection (y axis); (b) ^1^H NMR spectroscopic comparison of (6*E*)-5-hydroxydendrolasin (**7a**) (i) and (6*Z*)-5-hydroxydendrolasin (**7b**) (ii); × = water signals.

Racemic 5-hydroxydendrolasin (**7a/b**) was next subjected to HPLC (hexanes/EtOAc, 90:10) to provide the individual *E-* (**7a**) and *Z*-isomers (**7b**) that were each carefully characterised by ^1^H NMR and 1D nOe experiments. Assignment of the upfield signals corresponding to the three methyl groups attached to the double bonds (Figure 3) was made using HMBC and nOe data. The signal for CH_3_-14 in the *Z*-isomer resonated downfield (δ_H_ 1.74) compared to that in the *E*-isomer (δ_H_ 1.65) while the signal for H-5 appeared slightly upfield (δ_H_ 4.48) in the *Z*-isomer compared to that in its *E* counterpart (δ_H_ 4.51). 1D NOESY irradiation of the CH_3_-14 signal for the *Z*-isomer resulted in enhancement of the alkene signal at δ_H_ 5.24 (H-6), fully consistent with the *Z* configuration; there was no corresponding nOe between CH_3_-14 and H-6 in the *E*-isomer, although such data must be interpreted with care.

**Figure 4:**
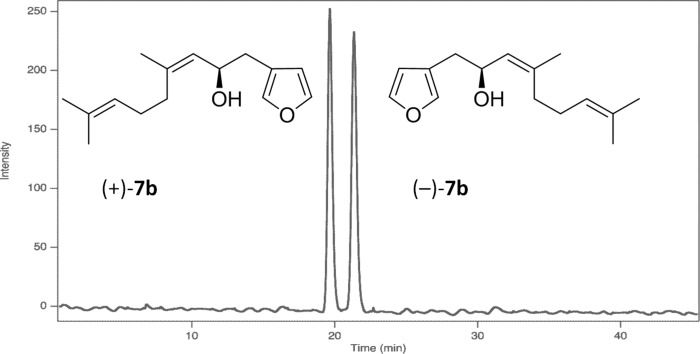
Analytical enantioselective HPLC trace showing separation of individual enantiomers of (6*Z*)-5-hydroxydendrolasin (Chiralpak AD column, 250 × 4.6 mm, 2% IPA in *n*-hexane at 0.5 mL/min, UV detection at 214 nm).

**Figure 5:**
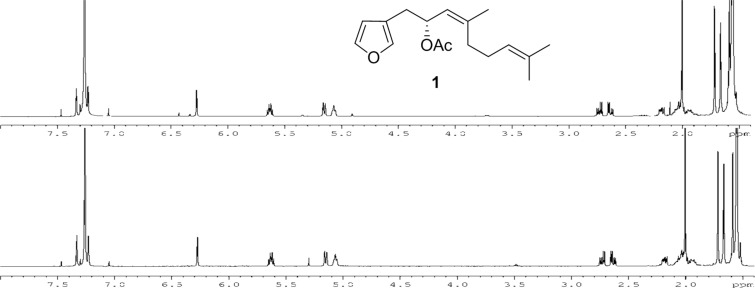
The ^1^H NMR (CDCl_3_, 500 MHz) spectroscopic comparison of (6*Z*)-dendrolasin-5-acetate (**1**): (a) sample isolated from *H. jacksoni*; (b) synthetic sample of (−)-(6*Z*)-**1**.

With the individual characterization of the *E*/Z-isomers accomplished, preparative enantioselective HPLC separation of racemic (*Z*)-5-hydroxydendrolasin (**7b**) was next undertaken (Figure 4) and provided samples of both of the (+)-**7b** and (−)-**7b** isomers, respectively. Treatment of each enantiomer of **7b** with acetic anhydride and pyridine gave their acetate derivatives. Surprisingly, (+)-**7b** ([α]_D_ +9.3) gave an acetate derivative with [α]_D_ −8.7 and vice versa ([α]_D_ –12 for (−)-**7b** and [α]_D_ +13 for its acetate derivative). As anticipated, the ^1^H NMR spectrum (CDCl_3_, 500 MHz) of the synthetic sample of (−)-(6*Z*)-**1** was fully superimposable with that of the sample of (6*Z*)-dendrolasin-5-acetate isolated from *H. jacksoni* (Figure 5).

### Absolute configurational assignment of (6*Z*)-dendrolasin-5-acetate (**1**)

**Figure 6:**
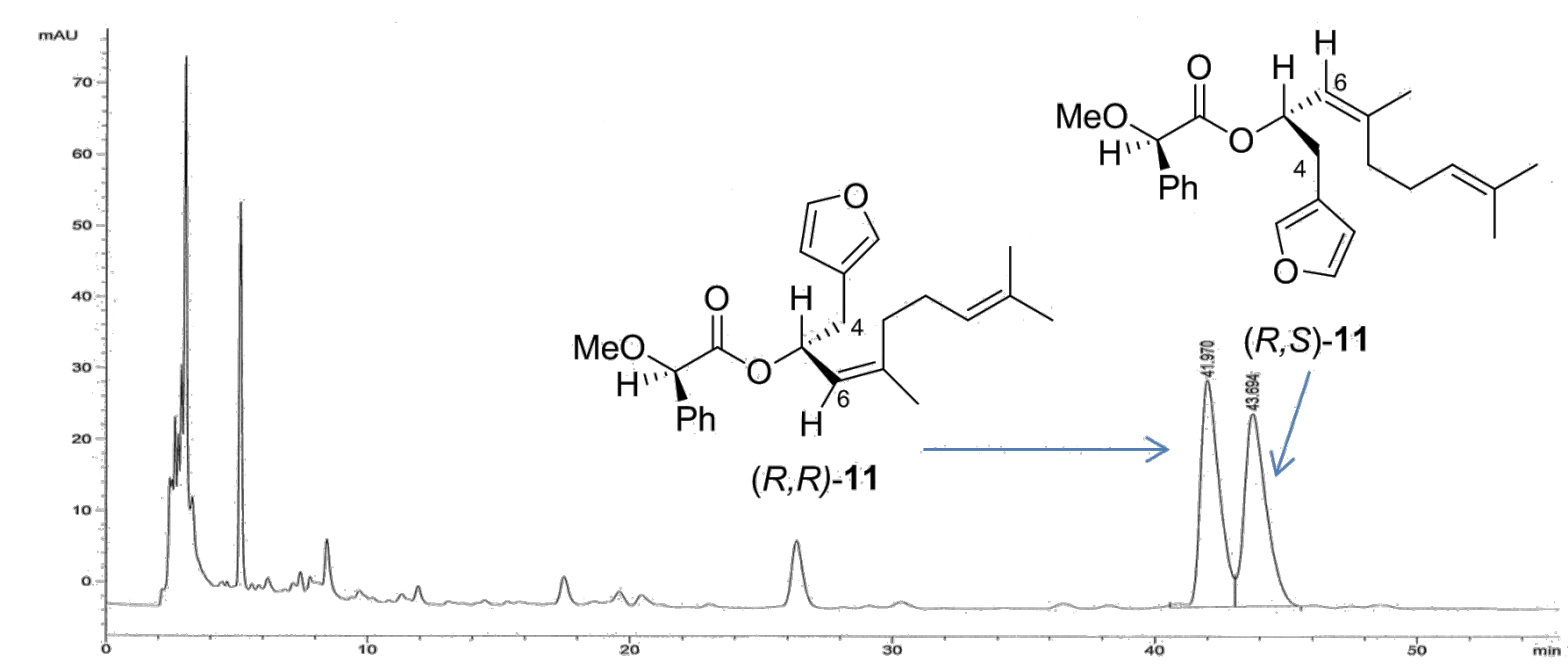
RP HPLC trace showing separation of the MPA esters (*R,R*)-**11** and (*R,S*)-**11** (MeCN/H_2_O, 63:35 over 50 minutes, Phenomenex C-18 column, 250 × 4.60 mm, UV detection at 254 nm).

It remained to determine the absolute configuration of C-5 in the natural acetate derivative. A portion of the racemic mixture of **7b** was esterified with (*R*)-MPA acid in the presence of DMAP and DCC at room temperature to give a mixture of diastereomeric (*R,R*)-**11** and (*R,S*)-**11**, which was separated by reversed phase (RP) HPLC (MeCN/H_2_O; 63:35) (Figure 6). The H-6 protons in (*R,R*)-**11** and (*R,S*)-**11** resonated at δ_H_ 5.04 and at δ_H_ 5.13, respectively, in agreement with H-6 of the (*R,R*)-isomer positioned in the shielding cone of the aryl ring of the MPA moiety [25]. The diastereotopic H-4 signals in (*R,S*)-**11** at δ_H_ 2.51 and 2.58 were shielded compared to those in (*R,R*)-**11** (δ_H_ 2.64, 2.74). Likewise, the furan proton signals were shifted upfield in the (*R,S*)-isomer compared to the (*R,R*)-isomer. These data were in agreement with the configurations depicted in Figure 6. The MPA ester spectra acted as reference data for determination of the absolute configuration of the natural sample of dendrolasin-5-acetate (**1**). The individually-purified enantiomers of synthetic (6*Z*)-5-hydroxydendrolasin were each treated with (*R*)-MPA to give their respective MPA ester derivatives. The ^1^H NMR spectra of the MPA ester of (+)-**7b** was identical to that of the synthetic (*R,R*)-**11** and established that (+)-5-hydroxydendrolasin (**7**) has an *R* configuration. Likewise, the ^1^H NMR spectrum of the MPA ester of (−)-**7b** was identical to that of the synthetic diastereomer (*R,S*)-**11**. Taking into account the change in sign of [α]_D_ between the alcohol and its acetate derivative, these data verified that the naturally-occurring (−)-acetate **1** had a 5*R* configuration.

**Figure 7:**
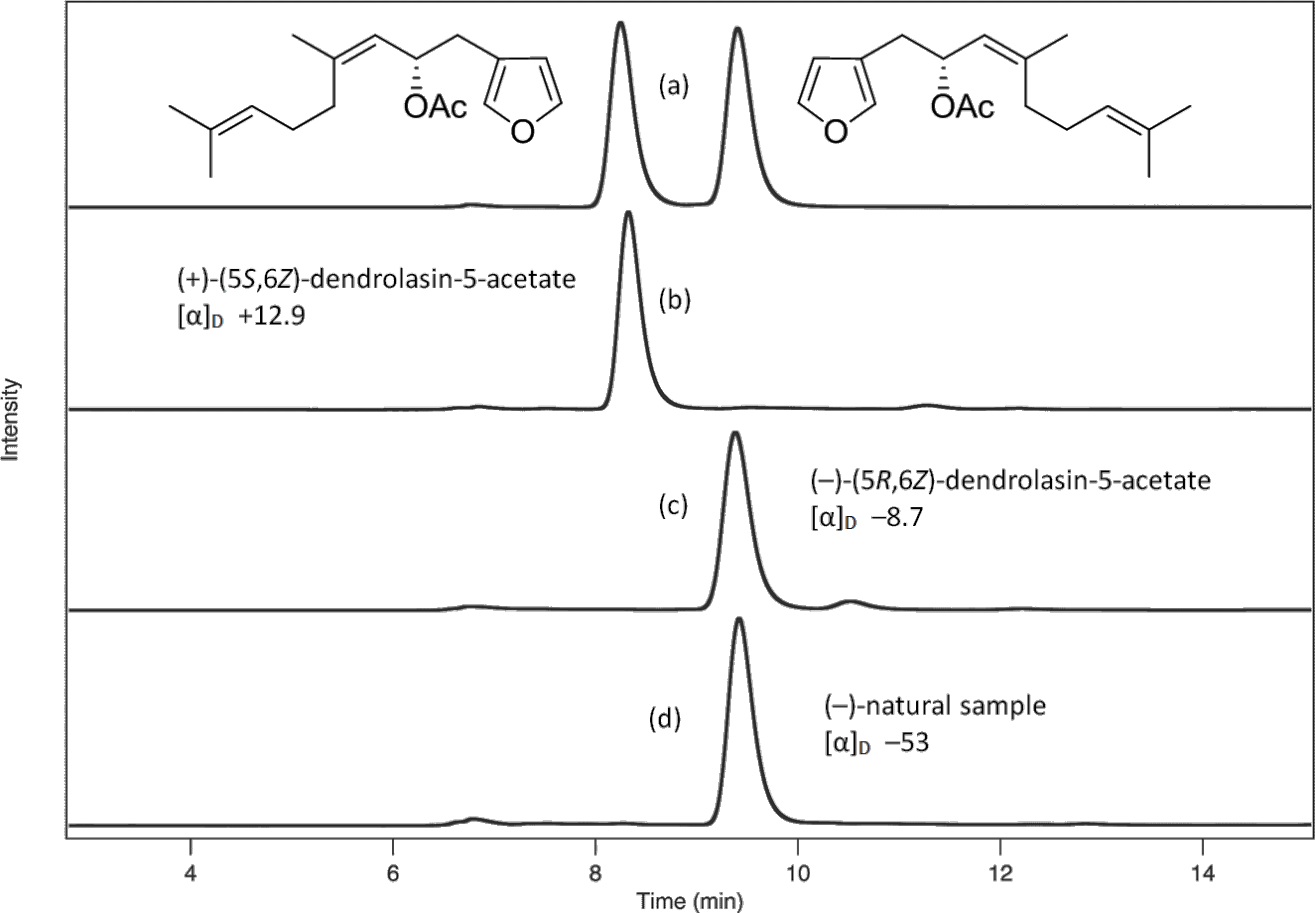
Enantioselective HPLC profiles (5% isopropanol in *n*-hexane) of **1**: (a) synthetic (±) mixture; (b) synthetic (+)-enantiomer; (c) synthetic (−)-enantiomer; (d) natural sample from *H. jacksoni*.

Specific rotation values for **1** were measured as [α]_D_ −53 for the natural sample and −8.7 for the synthetic sample. Despite having the same negative sign of specific optical rotation, the [α]_D_ values of the natural sample was considerably larger than that of the synthetic sample. Although a partial explanation for the difference in values was apparent, namely that the natural sample size was small (0.1 mg) compared to the synthetic sample size (0.9 mg), better experimental evidence of their stereochemical uniformity was desirable. Enantioselective HPLC analyses run under identical conditions using 5% isopropanol in *n*-hexane were carried out on the synthetic sample of racemic **1**, the two synthetically prepared *R* and *S* enantiomers of **1**, and the natural sample as shown in Figure 7. The data revealed a clear separation of the individual enantiomers of **1** (shown in trace (a)), and that the (+)-enantiomer elutes before the (−)-enantiomer (traces (b) and (c)). Finally, the natural sample has the same retention time as the (−)-enantiomer (trace (d)). A co-injection experiment performed on a mixture of the synthetic (+)-enantiomer and the natural sample consistently displayed two peaks in which the former compound eluted before the natural sample.

## Conclusion

Chemical investigation of *H. jacksoni* collected from Queensland yielded a new sesquiterpene (5*R*,6*Z*)-dendrolasin-5-acetate (**1**) whose structure and stereochemistry were confirmed by means of a five-step synthesis involving [1,2]-Wittig rearrangement of geranyl furyl ether (**6**) followed by acetylation of the purified alcohol products. The absolute configuration in **1** was established as 5*R* by detailed comparison with MPA ester derivatives prepared from individual enantiomers of synthetic (6*Z*)-5-hydroxydendrolasin purified by preparative enantioselective HPLC.

## Experimental

**Isolation of (−)-(5*R*,6*Z*)-dendrolasin-5-acetate (1) from *Hypselodoris jacksoni*:** Eight individuals of *H. jacksoni* (21–40 mm in length) were collected from various dive sites in South East Queensland (specimens #243, 244 Mudjimba Island, Mooloolaba S26°36’54”, E153°06’49”; #75–77 and #125–127 Shag Rock, North Stradbroke Island S27°24'49" E153°31'30”) between March and October of 2012. The whole animals were chopped, macerated in acetone (20 mL), sonicated for 2 min before being filtered through a cotton plug. The resulted filtrate was combined, concentrated under reduced pressure and partitioned between Et_2_O (3 × 20 mL) and water. The Et_2_O crude fraction (39.2 mg) was resolved by NP flash column chromatography eluted with gradient solvent from 100% hexanes→100% EtOAc to give ten fractions. The first and the second fraction (coded as HJF1–2) (21.3 mg) were combined based on their TLC profile, and yielded the major metabolites (−)-furodysinin (3.8 mg), (+)-agassizin (2.5 mg) and (−)-euryfuran (0.8 mg) following NP HPLC separation using 100% hexanes. The fourth fraction coded as HJF4 (1.8 mg) was subjected to NP HPLC eluting with 2.5% EtOAc/hexanes to give (−)-dehydroherbadysidolide (0.1 mg), (+)-pallescensone (0.2 mg), and the new sesquiterpene (−)-(5*R*,6*Z*)-dendrolasin-5-acetate (**1**, 0.1 mg). 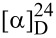 −53 (*c* 0.006, CHCl_3_); ^1^H NMR (CDCl_3_, 500 MHz) δ_H_ 7.33 (bs, 1H, H-1), 7.23 (bs, 1H, H-15), 6.27 (s, 1H, H-2), 5.64 (bdt, *J* = 6.4, 9.3 Hz, 1H, H-5), 5.15 (d, *J* = 9.3 Hz, 1H, H-6), 5.07 (brt, *J* = 6.9 Hz, 1H, H-10), 2.73 (dd, *J* = 6.7, 14.5 Hz, 1H, H-4a), 2.63 (dd, *J* = 6.0, 14.5 Hz, 1H, H-4b), 2.15–1.95 (overlapping, m, 4H, H-8, H-9), 1.72 (s, 3H, H-14), 1.67 (s, 3H, H-12), 1.59 (s, 3H, H-13); ^13^C NMR (CDCl_3_, 125 MHz) δ_C_ 142.7 (C-1), 141.5 (C-7), 140.1 (C-15), 132.0 (C-11), 123.9 (C-10), 123.7 (C-6), 120.2 (C-3), 111.8 (C-2), 70.7 (C-5), 32.5 (C-8), 30.7 (C-4), 26.5 (C-9), 25.7 (C-12), 23.3 (C-14), 17.6 (C-13); (+)-HRESIMS *m*/*z*: [M + Na]^+^ calcd. for C_17_H_24_NaO_3_, 299.1618; found, 299.1624.

**Procedure for Wittig rearrangement of 3-furylmethyl ether (6)** [21]: To a solution of (*E*/*Z*)-3-furylmethyl ether (**6**, 200 mg) in THF (10 mL) was added dropwise first LDA (3.64 mL, 10 wt % suspension in hexane) followed by *t*-BuLi (2.5 mL, 1.6 M in hexanes;) at −78 °C under Ar. After stirring for 1 h (the reaction mixture was allowed to warm to 0 °C), the reaction mixture was quenched with sat. aq NH_4_Cl solution and the solvent was removed under vacuum. The residue was extracted with pentane/Et_2_O (1:1, v/v). The extract was washed with brine and dried over Na_2_SO_4_. Evaporation of the solvent gave a residue, which was chromatographed on silica gel (5.0 g, hexanes/AcOEt 95:5) to afford the desired product (*E*/*Z* ratio ~3:1) as a mixture of enantiomers (83 mg, 39% yield). The *E-* and *Z-*isomers of 5-hydroxydendrolasin were then separated by NP HPLC (hexanes/EtOAc 90:10, flow rate 2 mL/min, refractive index detection, Waters μPorasil^TM^ column 10 μm, 300 × 7.8 mm) to give the individual 6*E* ((**7a**); 25.2 mg; *t*_R_ = 50.0 min) and 6*Z* ((**7b**); 8.4 mg; *t*_R_ = 40.0 min) isomers. Analytical enantioselective HPLC separation of the *Z*-isomer of 5-hydroxydendrolasin (**7b**) was performed on an Agilent 1200 series liquid chromatography system (isocratic conditions of 2% isopropanol in *n*-hexane, flow rate of 0.5 mL min^−1^) equipped with UV (214 nm) and ALP (Advanced Laser Polarimeter, PDR-Chiral Inc.) detectors and a Chiralpak AD column (250 × 4.6 mm, Daicel Chemical Industries Ltd.). The preparative enantioselective HPLC purification of **7b** used isocratic conditions (1.5% isopropanol in *n*-hexane, flow rate of 10 mL min^−1^) with UV detection at 220 nm and a Chiralpak AD column (250 × 20 mm, Daicel Chemical Industries Ltd.) to give the (+)-enantiomer (2.7 mg); (−)-enantiomer (2.6 mg). The retention times were: 29.5 min ((+)-enantiomer); 32.1 min ((−)-enantiomer).

**(±)-(*E*)-1-(furan-3-yl)-4,8-dimethylnona-3,7-dien-2-ol (7a)** [21]**:** Colorless glass; ^1^H NMR (CDCl_3_, 500 MHz) δ_H_ 7.37 (s, 1H, H-1), 7.29 (s, 1H, H-15), 6.32 (s, 1H, H-2), 5.24 (d, *J* = 8.5 Hz, 1H, H-6), 5.07 (brt, *J* = 7.1 Hz, 1H, H-10), 4.51 (q, *J* = 7.1, 13.6 Hz, 1H, H-5), 2.66 (dd, *J* = 7.1, 14.4 Hz, 1H, H-4a), 2.59 (dd, *J* = 7.1, 14.4 Hz, 1H, H-4b), 2.12–2.01 (m, 4H, H-8 and H-9), 1.65 (d, *J* = 1.1 Hz, 3H, CH_3_-14), 1.69 (s, 3H, CH_3_-12), 1.60 (s, 3H, CH_3_-13); ^13^C NMR (CDCl_3_, 125 MHz) δ_C_ 142.9 (C-1), 140.3 (C-15), 139.5 (C-7), 131.8 (C-11), 127.0 (C-3), 124.0 (C-10), 120.9 (C-6), 111.7 (C-2), 68.5 (C-5), 39.6 (C-8), 33.3 (C-4), 26.4 (C-9), 25.7 (C-12), 17.8 (C-13), 16.7 (C-14); (+)-LRESIMS *m*/*z*: 257 [M + Na]^+^.

**(±)-(*Z*)-1-(furan-3-yl)-4,8-dimethylnona-3,7-dien-2-ol (7b):** Colorless glass; ^1^H NMR (CDCl_3_, 500 MHz) δ_H_ 7.37 (s, 1H, H-1), 7.29 (s, 1H, H-15), 6.32 (s, 1H, H-2), 5.24 (d, *J* = 8.8 Hz, 1H, H-6), 5.07 (brt, *J* = 5.4 Hz, 1H, H-10), 4.48 (q, *J* = 6.7, 13.6 Hz, 1H, H-5), 2.65 (dd, *J* = 7.2, 14.4 Hz, 1H, H-4a), 2.58 (dd, *J* = 7.2, 14.4 Hz, 1H, H-4b), 2.12–2.01 (m, 4H, H-8 and H-9), 1.74 (d, *J* = 1.1 Hz, 3H, CH_3_-14), 1.69 (s, 3H, CH_3_-12), 1.60 (s, 3H, CH_3_-13); ^13^C NMR (CDCl_3_, 125 MHz) δ_C_ 142.9 (C-1), 140.3 (C-15), 139.5 (C-7), 132.6 (C-11), 128.0 (C-3), 124.0 (C-10), 121.1 (C-6), 111.7 (C-2), 68.1 (C-5), 33.2 (C-8), 32.5 (C-4), 26.6 (C-9), 25.8 (C-12), 23.5 (C-14), 17.8 (C-13); (+)-HRESIMS *m*/*z*: [M + Na]^+^ calcd for C_15_H_22_NaO_2_, 257.1512; found, 257.1515.

**Procedure for acetylation of (±)-(*Z*)-1-(furan-3-yl)-4,8-dimethylnona-3,7-dien-2-ol (7b):** A portion of **7b** (8.4 mg) was dissolved in dry pyridine (0.5 mL) and cooled in an ice bath. After the addition of acetic acid anhydride (1.5 mL) the mixture brought to rt and stirred for 1.5 hours. Toluene (0.5 mL) was added and the resulting azeotrope was evaporated under reduced pressure to give (**±**)-(6*Z*)-dendrolasin-5-acetate (**1**) (6.4 mg); ^1^H and ^13^C NMR data (CDCl_3_, 500 MHz) are identical to those of **1**. (+)-LRESIMS *m/z* 299 [M + Na]^+^.

**Procedure for acetylation of the (+) isomer of 7b:** A portion of the (+) isomer of **7b** (1.3 mg) was acetylated using the same procedure as for **7b** to obtain (**−**)-(6*Z*)-dendrolasin-5-acetate (**1**, 0.9 mg); Colorless glass; [α]_D_ −8.7 (*c* 0.06, CHCl_3_); ^1^H and ^13^C NMR data (CDCl_3_, 500 MHz) are the same as those of the **1**. (+)-LRESIMS *m*/*z*: 299 [M + Na]^+^.

**Procedure for acetylation of the (−)-isomer of 7b:** A portion of the (**−**)-isomer of **7b** (1.3 mg) was acetylated using the same procedure as for **7b** to obtain (**+**)-(6Z)-dendrolasin-5-acetate (**1**, 1.1 mg); Colorless glass; [α]_D_ +12.9 (*c* 0.07, CHCl_3_); ^1^H and ^13^C NMR (CDCl_3_, 500 MHz) are identical to those of **1**. (+)-LRESIMS *m*/*z*: 299 [M + Na]^+^.

**Procedure for preparation of (*R*)- and (*S*)-MPA esters of racemic (6*Z*)-5-hydroxydendrolasin (7b):** (**±**)-(6*Z*)-5-Hydroxydendrolasin (2.0 mg) was dissolved in dry DCM (0.2 mL) to which (*R*)-methoxyphenylacetic acid (MPA) (0.83 mg in 0.1 mL of DCM) was added, followed by DCC (2 equiv, 1.03 mg in 0.1 mL of DCM) and DMAP (2 equiv, 0.61 mg in 0.1 mL of DCM). The reaction was stirred at rt for 16 h and then filtered through a cotton wool plug. The solvent was removed by rotary evaporation and the residue passed through a pipette silica column eluted with hexanes/EtOAc (95:5) to give a racemic MPA ester (0.8 mg). The racemic MPA ester was subsequently subjected to RP HPLC (MeCN/H_2_O, 60:40; flow rate 1 mL/min, UV detection at 254 nm, Phenomenex Gemini 5 μ C18 110 Å, 250 × 4.60 mm) to give the *R,R* (0.2 mg) and *R,S* diastereomers (0.2 mg).

**(*R,R*)-(6*Z*)-dendrolasin MPA ester (11a):** Colorless glass; [α]_D_ +10.1 (*c* 0.01, CHCl_3_); ^1^H NMR (CDCl_3_, 500 MHz) δ_H_ 7.38–7.30 (m, 5H, MPA phenyl protons), 7.28 (brs, 1H, H-1), 7.14 (s, 1H, H-15), 6.20 (s, 1H, H-2), 5.66 (m, 1H, H-5), 5.04 (d, *J* = 9.8 Hz, 1H, H-6), 5.02 (brt, 1H, H-10), 4.70 (s, 1H, C*H* of MPA), 3.37 (s, 3H, OMe of MPA), 2.73 (dd, *J* = 7.2, 14.7 Hz, 1H, H-4a), 2.63 (dd, *J* = 7.2, 14.7 Hz, 1H, H-4b), 2.16–2.10 (m, 1H, H-8a), 1.98–1.86 (m, 1H, H-8b and 2H, m, H-9), 1.64 (s, 6H, CH_3_-14 and CH_3_-12), 1.56 (s, 3H, CH_3_-13); (+)-HRESIMS *m*/*z*: [M + Na]^+^ calcd for C_24_H_30_NaO_4_, 405.2036; found, 405.2038.

**(*R,S*)-(6*Z*)-dendrolasin MPA ester (11b):** Colorless glass; [α]_D_ +10.7 (*c* 0.01, CHCl_3_); ^1^H NMR (CDCl_3_, 500 MHz) δ_H_ 7.38−7.36 (m, 5H, MPA phenyl protons), 7.17 (bs, 1H, H-1), 6.83 (s, 1H, H-15), 5.95 (s, 1H, H-2), 5.68 (m, 1H, H-5), 5.12 (d, *J* = 9.4 Hz, 1H, H-6), 5.04 (brt, *J* = 7.3 Hz, 1H, H-10), 4.68 (s, 1H, C*H* of MPA), 3.36 (s, 3H, OMe of MPA), 2.58 (dd, *J* = 7.0, 14.7 Hz, 1H, H-4a), 2.51 (dd, *J* = 7.0, 14.7 Hz, 1H, H-4b), 2.24–2.20 (m, 1H, H-8a), 2.06–1.92 (m, 1H, H-8b and m, 2H, H-9), 1.71 (d, *J* = 1.0 Hz, 3H, CH_3_-14), 1.67 (s, 3H, CH_3_-12), 1.58 (m, 3H, CH_3_-13); (+)-HRESIMS *m*/*z*: [M + Na]^+^ calcd for C_24_H_30_NaO_4_, 405.2036; found, 405.2043.

**Procedure for preparation of MPA ester of the (+)-isomer of 7b:** A portion of the (+)-isomer of **7b** (1.3 mg) was treated with (*R*)-MPA (0.83 mg in 0.1 mL of DCM) using the same procedure as for the preparation of MPA ester of the racemic (6*Z*)-5-hydroxydendrolasin (**7b**) to obtain the (+)-MPA ester (1.2 mg). The ^1^H NMR data (CDCl_3_, 500 MHz) were identical to those for the (*R,R*,6*Z*)-dendrolasin MPA ester (**11a**). [α]_D_ −93 (*c* 0.08, CHCl_3_); (+)-LRESIMS *m*/*z*: 405 [M + Na]^+^.

**Procedure for preparation of MPA ester of the (−)-isomer of 7b:** A portion of the (−)-isomer of **7b** (1.3 mg) was treated with (*R*)-MPA (0.83 mg in 0.1 mL of DCM) using the same procedure as for the preparation of MPA ester of the racemic (6*Z*)-5-hydroxydendrolasin (**7**) to obtain the (−)-MPA ester (1.0 mg). The ^1^H NMR data (CDCl_3_, 500 MHz) are identical to those of the (*R,S*,6*Z*)-dendrolasin MPA ester (**11b**). [α]_D_ −22 (*c* 0.07, CHCl_3_); (+)-LRESIMS *m*/*z*: 405 [M + Na]^+^.

**Enantioselective HPLC experiments:** Enantioselective HPLC analysis of the (±)-(6*Z*)-dendrolasin-5-acetate (**1**) racemic mixture, the individual 5*R-* and 5*S*-enantiomers of (6*Z*)-dendrolasin-5-acetate, and the natural sample of (−)-(5*R*,6Z)-dendrolasin-5-acetate, were performed using an Agilent 1200 series liquid chromatography system (isocratic conditions, 5% isopropanol in *n*-hexane, flow rate of 0.5 mL min^−1^) equipped with UV (220 nm) and ALP (Advanced Laser Polarimeter, PDR-Chiral Inc.) detectors and a Chiralpak OJ column (4.6 × 250 mm, Daicel Chemical Industries Ltd.). Retention times were: (+)-enantiomer, >97% ee 8.3 min; (–)-enantiomer, >98.5% ee 9.4 min; natural sample 9.4 min.

## Supporting Information

Supporting Information File 1 contains experimental details for the preparation of compound **6**, spectroscopic data and other relevant information for compounds **1**, **7b**, **11a**, and **11b**.

File 1 Experimental details and spectroscopic data.
