# Supplementary material for: Synthesis and determination of the absolute configuration of (−)-(5R,6Z)-dendrolasin-5-acetate from the nudibranch Hypselodoris jacksoni
Source: Beilstein J Org Chem. 2013 Dec 23;9:2925–33. doi: 10.3762/bjoc.9.329 (PMC3896254; doi:10.3762/bjoc.9.329)

Supporting Information

for

Synthesis and determination of the absolute configuration of (−)-(5*R*,6*Z*)-dendrolasin-5-acetate from the nudibranch *Hypselodoris jacksoni*

I. Wayan Mudianta[1], Victoria L. Challinor[1], Anne E. Winters[2], Karen L. Cheney[2], James J. De Voss[1] and Mary J. Garson[1*]

Address: [1] School of Chemistry and Molecular Biosciences, The University of Queensland, Brisbane QLD 4072 Australia and [2] School of Biological Sciences, The University of Queensland, Brisbane QLD 4072 Australia

Email: Mary J. Garson - m.garson@uq.edu.au
*Corresponding author

Experimental details and spectroscopic data

S1: Procedure for etherification of 3-furanmethanol…………………………………………… S2

S2: The ^{1}H NMR spectrum ($CDCl_3$, 500 MHz) of natural sample (−),(5*R*,6*Z*)-dendrolasin-5-acetate (**1**) ……………………………………………..………………………………… S3

S3: gCOSY spectrum ($CDCl_3$, 500 MHz) of **1**……………………………………………….. S3

S4: gHSQC spectrum ($CDCl_3$, 500 MHz) of **1**………………………………………………. S4

S5: gHMBC spectrum ($CDCl_3$, 500 MHz) of **1**………………………………………………. S4

S6: The ^{1}H NMR spectrum ($CDCl_3$, 500 MHz) of (±)-(*E*)-1-(furan-3-yl)-4,8-dimethylnona-3,7-dien-2-ol (**7a**)…………………………………………………………………………….. S5

S7: The ^{1}H NMR spectrum ($CDCl_3$, 500 MHz) of (±)-(*Z*)-1-(furan-3-yl)-4,8-dimethylnona-3,7-dien-2-ol (**7b**) ……………………………………………………………………………. S5

S8: The ^{1}H NMR spectrum ($CDCl_3$, 500 MHz) of (*R*,*R*)-(6*Z*)-dendrolasin MPA ester (**11a**)…. S6

S9: The ^{1}H NMR spectrum ($CDCl_3$, 500 MHz) of (*R*,*S*)-(6*Z*)-dendrolasin MPA ester (**11b**)….. S6

S1: Procedure for etherification of 3-furanmethanol [1]

To a solution of geranyl bromide (**5**, 499.1 mg, 2.3 mmol) containing neryl bromide (<5%) and 3-furylmethanol [2] (125 mg, 1.3 mmol) (**4**) in DMF (3 mL) was added NaH (ca. 60 % purity, 91.9 mg, ca. 36.7 mmol) at 0 °C and stirring was continued for 8 h at rt. The reaction was carefully quenched with sat. aq NH_4Cl solution in an ice bath. The reaction was extracted with Et_2O and CH_2Cl_2 (v/v, 2:1), and the combined organic layers were washed with brine. The organic layer was dried over Na_2SO_4 and evaporated to give a residue, which was chromatographed on silica gel (80 g, hexanes/Et_2O = 95:5) to afford an *E*/*Z* mixture (3:1) of the 3-furylmethyl ether (**6**, 386.5 mg) as a yellowish oil.

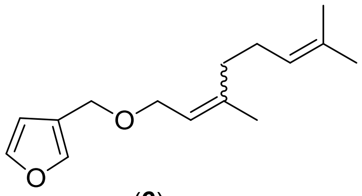

(**6**)

Geranyl 3-furylmethyl ether (**6**): ^{1}H-NMR ($CDCl_3$, 500 MHz): δ_H 7.39 (1H, s, H-1), 7.37 (1H, s, H-15), 6.42 (1H, s, H-2), 5.37 (1H, t, *J* = 6.7, H-6), 5.09 (1H, t, *J* = 6.7, H-10), 4.37 (2H, s, H-4), 4.00 (2H, d, *J* = 6.8 Hz, H-5), 2.10 (2H, m, H-8), 2.04 (2H, m, H-9), 1.67 (3H, s, CH_3-14), 1.65 (3H, s, CH_3-12), 1.59 (3H, s, CH_3-13). (+)-LRESIMS *m/z* 234.33 $[M + Na]^+$.

References

[1] Tsubuki, M.; Okita, H.; Kaneko, K.; Shigihara, A.; Honda, T. *Heterocycles* **2009**, *77*, 433-444

[2] Wang, E. S.; Choy, Y. M.; Wong, H. N. C. *Tetrahedron* **1996**, *52*, 12137-12158.

S2: The ^{1}H NMR spectrum ($CDCl_3$, 500 MHz) of the natural sample of (−)-(5*R*,6*Z*)-dendrolasin-5-acetate (1)

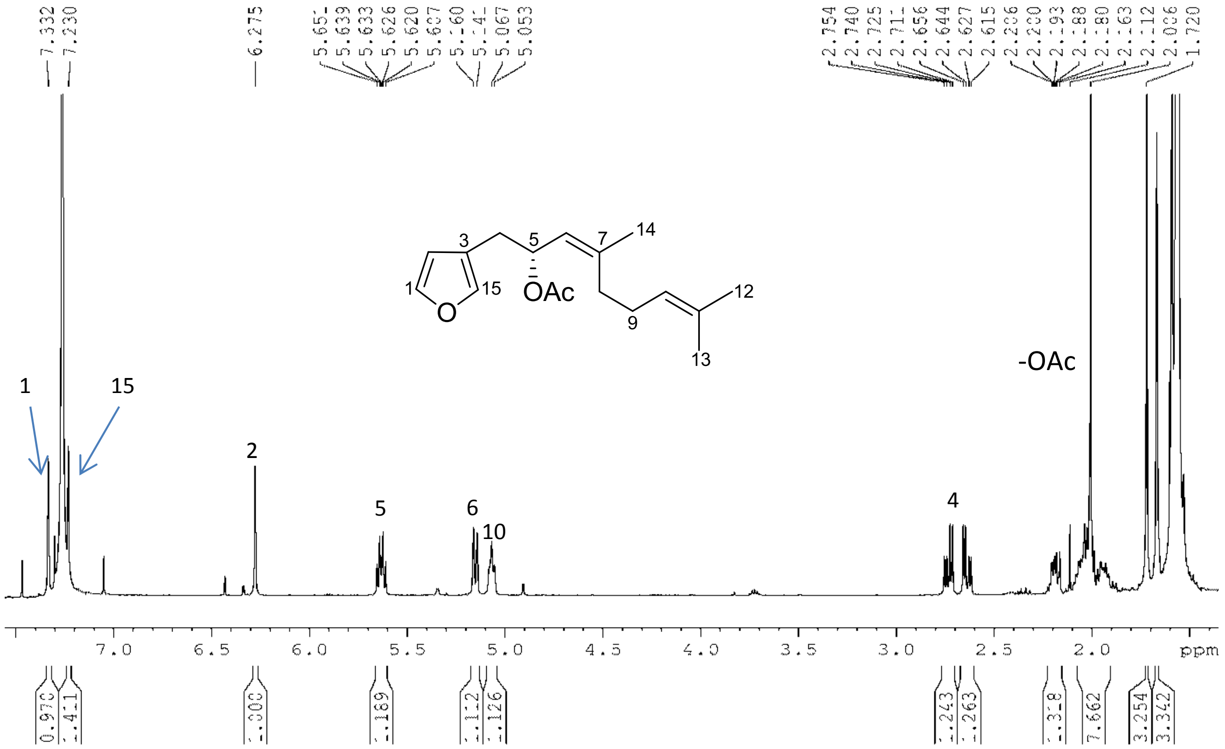

S3: gCOSY spectrum ($CDCl_3$, 500 MHz) of 1

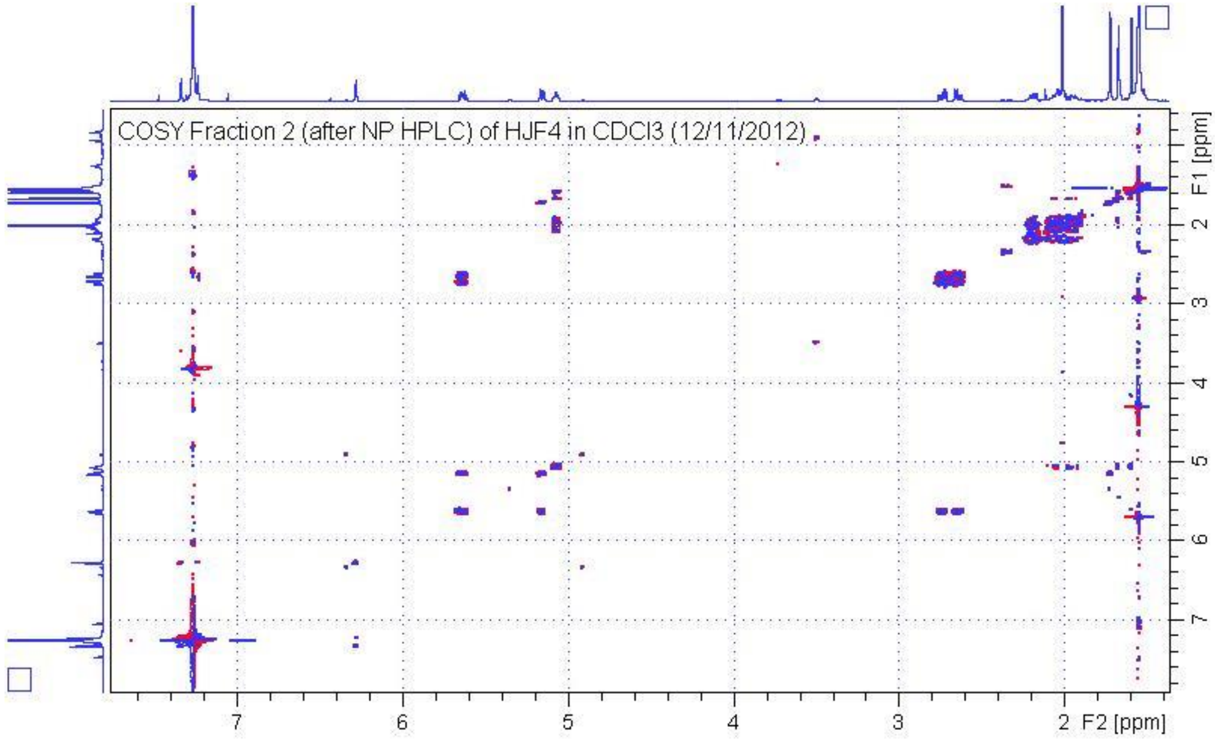

S4: gHSQC spectrum ($CDCl_3$, 500 MHz) of 1

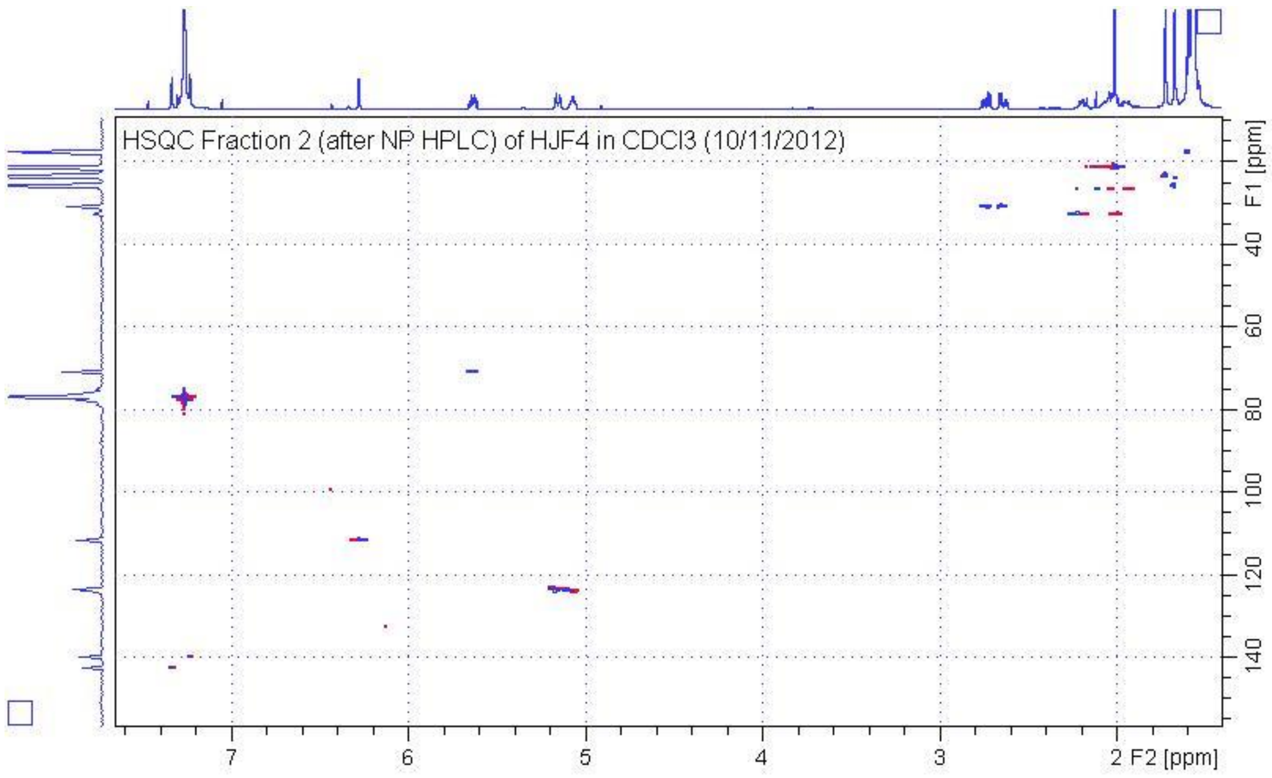

S5: gHMBC spectrum ($CDCl_3$, 500 MHz) of 1

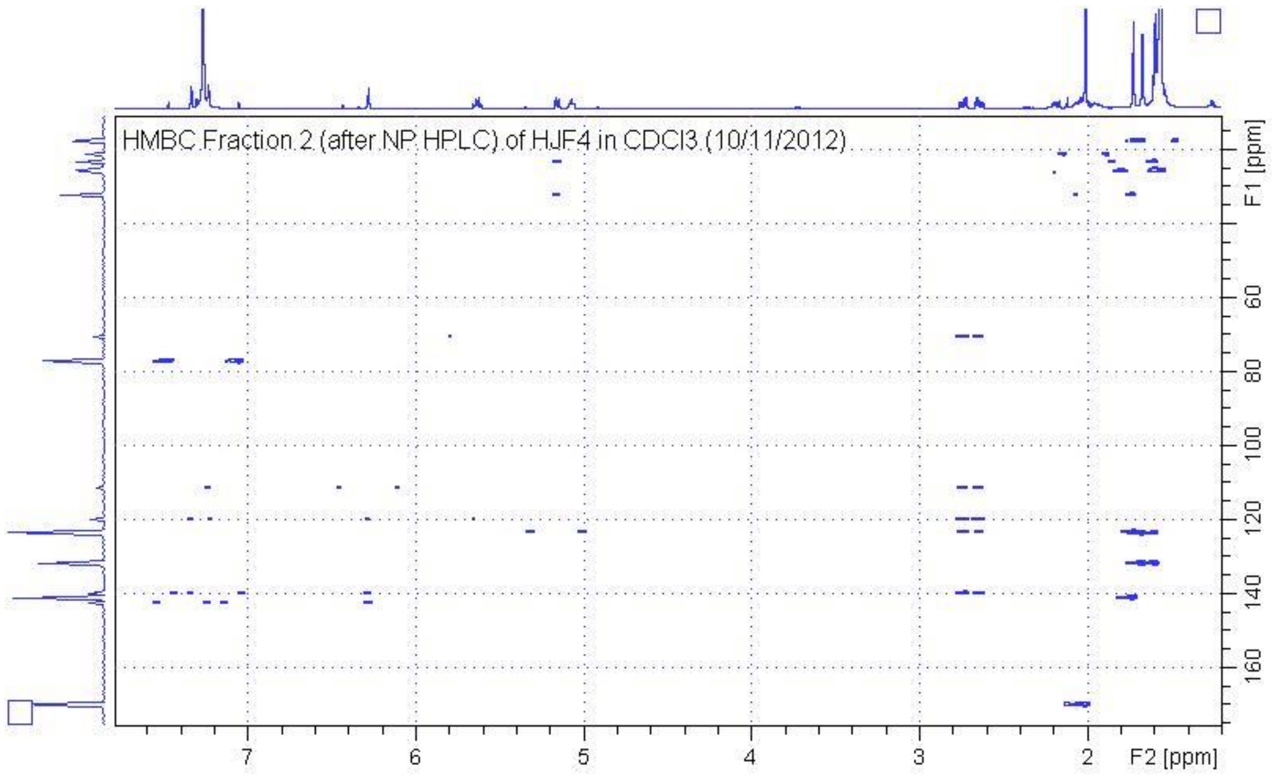

S6: The ^{1}H NMR spectrum ($CDCl_3$, 500 MHz) of (±)-(*E*)-1-(furan-3-yl)-4,8-dimethylnona-3,7-dien-2-ol (7a)

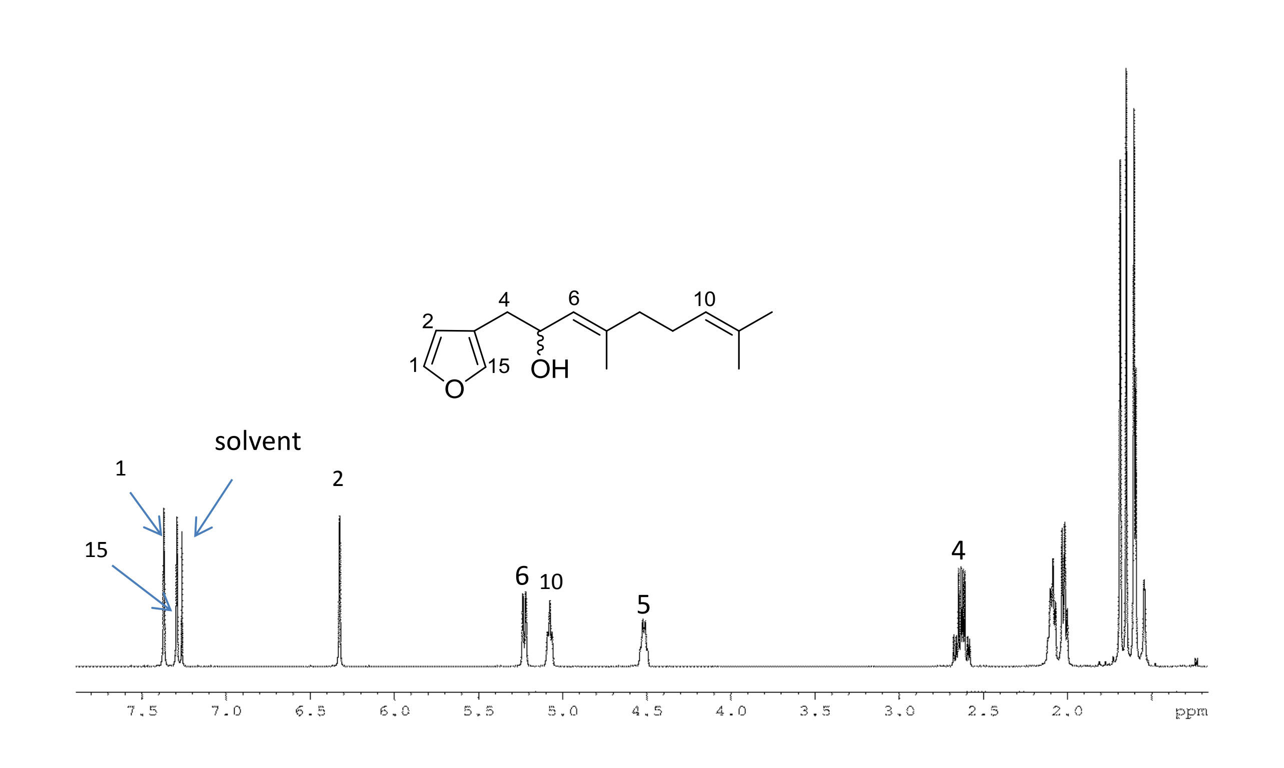

S7: The ^{1}H NMR spectrum ($CDCl_3$, 500 MHz) of (±)-(*Z*)-1-(furan-3-yl)-4,8-dimethylnona-3,7-dien-2-ol (7b)

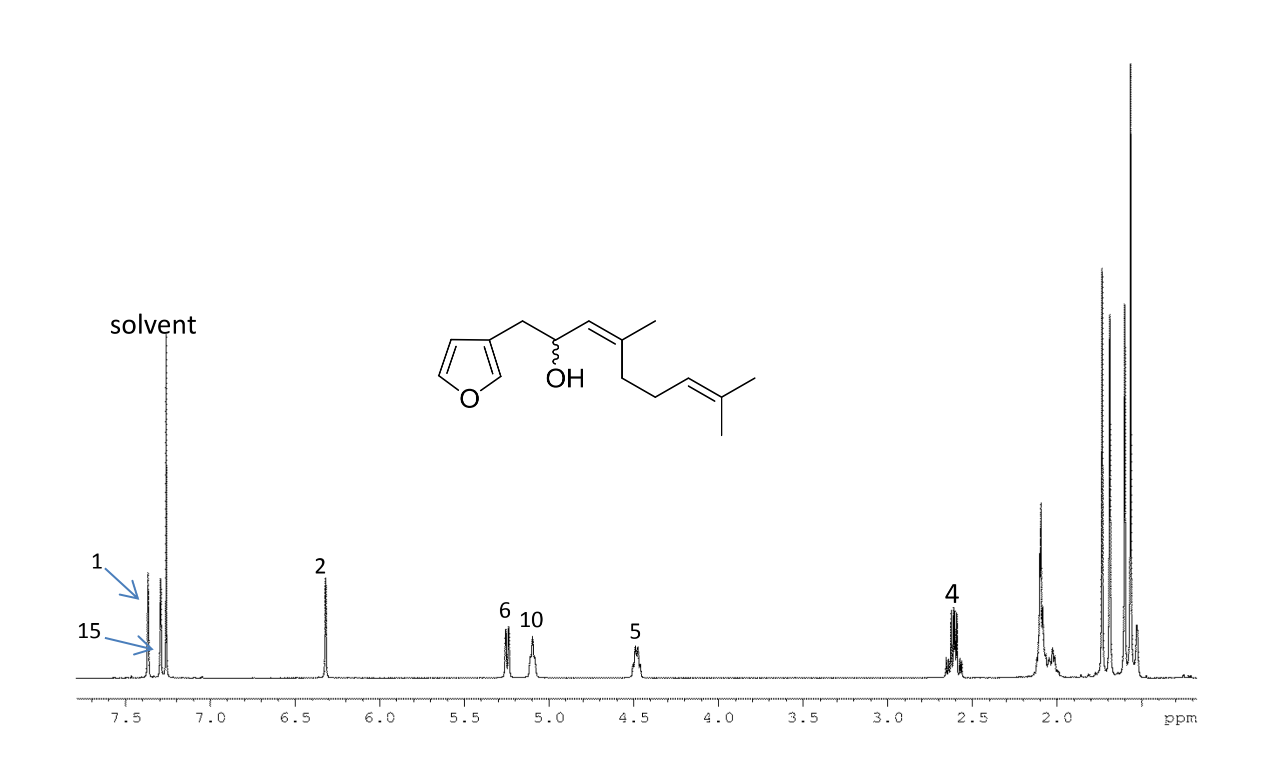

S8: The ^{1}H NMR spectrum ($CDCl_3$, 500 MHz) of (*R*,*R*)-(6*Z*)-dendrolasin MPA ester (11a)

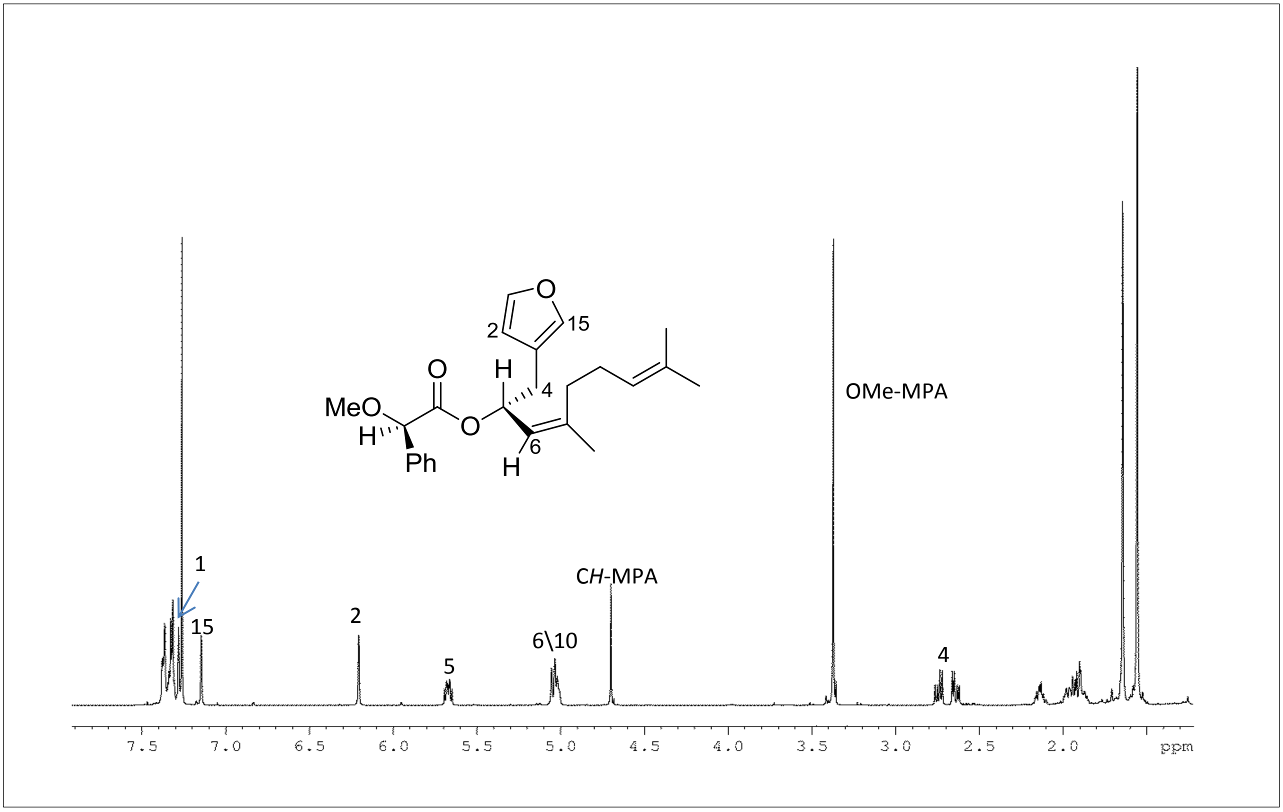

S9: The ^{1}H NMR spectrum ($CDCl_3$, 500 MHz) of (*R*,*S*)-(6*Z*)-dendrolasin MPA ester (11b)

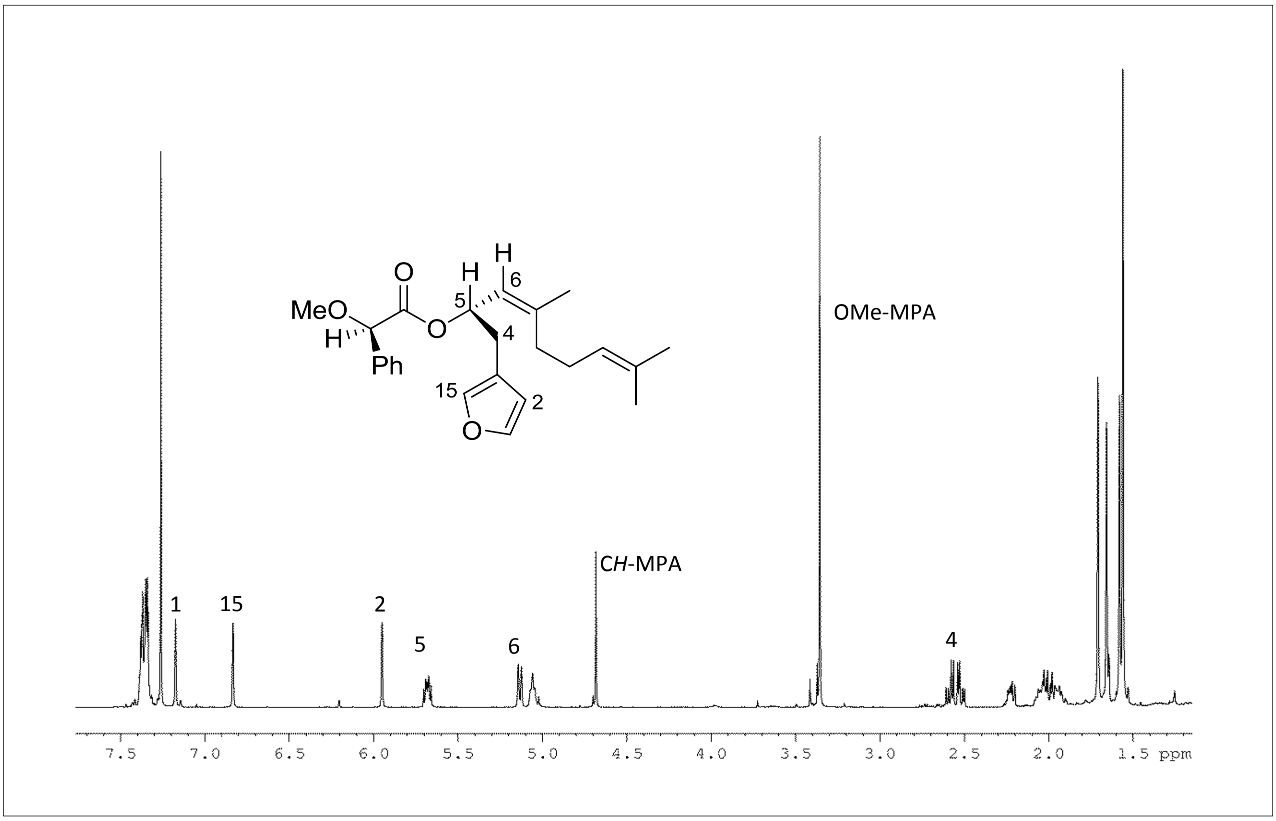

Supplement: File 1 — Experimental details and spectroscopic data. [file Beilstein_J_Org_Chem-09-2925-s001.pdf]
